# New onset diabetes complicated by haemolysis and rhabdomyolysis: a case report and review of the literature

**DOI:** 10.1186/1752-1947-2-159

**Published:** 2008-05-16

**Authors:** Clare M Galtrey, Rohan Pathansali

**Affiliations:** 1 Department of Medicine, Kings College Hospital, Denmark Hill, London, SE5 9RS, UK

## Abstract

**Introduction:**

Previously undiagnosed glucose-6-phosphate dehydrogenase (G6PD) deficiency can be unmasked by a diabetic crisis and both can be associated with rhabdomyolysis. The relationship between diabetes and G6PD deficiency is discussed and the possible triggers for haemolysis as outlined in this case report. The incidence of G6PD deficiency is 10% in African-American males and up to 35% in parts of Africa so an increased awareness of G6PD deficiency is important when treating diabetes in these populations.

**Case presentation:**

A 54-year-old Kenyan man presented with a 3-day history of reduced appetite, weakness and reduced level of consciousness as a result of a hyperglycaemic diabetic crisis with both hyperosmolarity and ketoacidosis. The patient then developed haemolysis and a raised creatine kinase level. A diagnosis of G6PD deficiency and rhabdomyolysis was made.

**Conclusion:**

This case highlights the importance of simple laboratory investigations in the early identification of the rarer complications of diabetic crisis such as haemolysis secondary to G6PD deficiency and rhabdomyolysis.

## Introduction

The acute hyperglycaemic complications of diabetes include diabetic ketoacidosis (DKA) and hyperosmolar nonketotic syndrome (HONK). Both are potentially life-threatening and complications include: ischaemia or infarction affecting any organ, particularly myocardial or cerebral; thromboembolic disease; acute respiratory distress syndrome; disseminated intravascular coagulation; multi-organ failure; rhabdomyolysis; cerebral oedema (rare in adults, less so in children); and iatrogenic complications due to inexpert rehydration and electrolyte management, over-administration of insulin or fluid overload leading to cardiac failure. Mortality from HONK is high, with reported death rates as high as 30% to 35%. HONK is the first manifestation of type 2 diabetes in about one-third of cases.

Glucose-6-phosphate dehydrogenase (G6PD) deficiency is an X-linked inherited disorder that increases the vulnerability of erythrocytes to oxidative stress and is the most common enzyme deficiency worldwide and usually affects persons of African, Asian, Mediterranean or Middle-Eastern descent. Different gene mutations cause different levels of enzyme deficiency, with classes assigned to various degrees of deficiency and disease manifestation. The two most common mutations are G6PD Mediterranean and G6PD African. For example, 10% of black males in the United States have moderate (10% to 60% of normal) enzyme activity. Complications include acute haemolysis and neonatal jaundice. This case demonstrates several complications that can occur with a new presentation of diabetes mellitus and highlights the importance of simple laboratory investigations including tests for methaemoglobinaemia and creatine kinase in the early identification of G6PD deficiency and rhabdomyolysis.

## Case presentation

A 54-year-old Kenyan man presented with a 3-day history of reduced appetite, weakness and reduced level of consciousness. There was no history of diabetes. On examination, blood pressure was 99/48 mmHg, pulse 121 beats per minute, respiratory rate 32 breaths per minute and temperature 37.5°C with a Glasgow Coma Score of 10/15. Laboratory investigations revealed: pH 7.172; 1+ urinary ketones; glucose 83.4 mmol/l (1502 mg/dl); HbA1c 5.6%; sodium 152 mmol/l; potassium 4.5 mmol/l; creatinine 384 μmol/l (4.34 mg/dl); urea 25 mmol/l (70 mg/dl); C-reactive protein (CRP) <5.0 mg/l; haemoglobin 18.6 g/dl; and white blood cell count 15.08 × 10^9^/litre.

Initially the patient was treated as diabetic ketoacidosis with intravenous insulin at a rate of 6 U/hour with 0.9% saline. Later, the plasma osmolality was calculated as 422 mmol/l and the diagnosis revised to diabetes mellitus with hyperosmolarity and acidosis. The rate of intravenous insulin was reduced to 3 U/hour and intravenous fluids changed to alternate 5% dextrose and 0.9% saline. Despite this, the next day the patient deteriorated further with quadriparesis, loss of speech and dysphagia and was transferred to a high-dependency unit with a presumed diagnosis of central pontine myelinolysis. Over the next 2 days plasma electrolytes and renal function normalised but CRP increased to 350 mg/l and ceftriaxone and metronidazole were started for presumed aspiration pneumonia. Clinically the patient improved and began speaking and eating. MRI scanning showed no evidence of pontine changes. After 3 days the patient was returned to the ward and the regimen was converted to oral gliclazide 160 mg BD.

**Figure 1:**
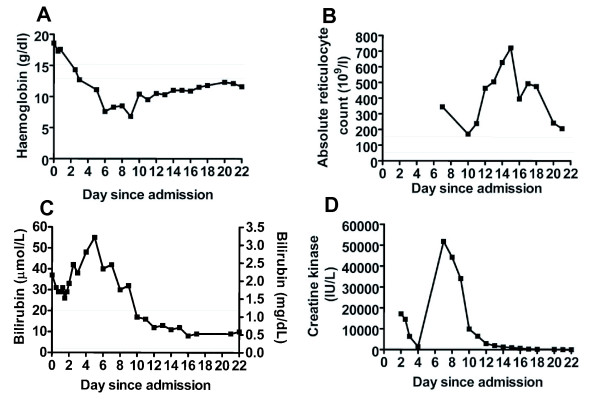
Laboratory investigations during the 22 days of admission. Normal values are shown by the grey lines. (A) Haemoglobin; (B) absolute reticulocyte count; (C) bilirubin; (D) creatine kinase.

The next day he became unwell again with an oxygen saturation on pulse oximetry of 85% that did not alter with the concentration of inspired oxygen, and with discoloured urine and muscle weakness. Despite the pulse oximetry reading, arterial blood gas showed a pO_2 _of 18.3 kPa but with a methaemoglobin level of 7.8%. Laboratory investigations revealed: bilirubin 48 μmol/l; phosphate 0.25 mmol/l; haemoglobin 7.6 g/dl; platelets 206 × 10^9^/litre. In view of the dropping haemoglobin and discoloured urine, a haemolysis screen and a creatine kinase level measurement were performed, which revealed the following results: LDH 3392 IU/l; haptoglobin 0.2 g/l; absolute reticulocyte count 345.6 × 10^9^/litre; negative direct antiglobulin test negative; and creatine kinase 51,800 IU/l (Figure 1). In the presence of the haemolysis and methaemoglobinaemia, G6PD and pyruvate kinase assays were performed. A diagnosis of G6PD deficiency 3.9 IU/gHb (5.2 to 11.5) and low pyruvate kinase 10.2 IU/gHb (11 to 19) was made. Rhabdomyolysis was also diagnosed. Retrospective creatine kinase levels were measured, which showed that it had been raised to a lesser degree earlier during his period of limb weakness (Figure 1D). His phosphate level was also low throughout this period but his renal function had remained within the normal range (Additional file 1).

The patient was treated with supportive blood transfusions and all medication was stopped apart from the intravenous insulin and intravenous fluids. His haemoglobin increased, reticulocytes count rose and serum creatine kinase and bilirubin returned to normal (Figure 1) and he was discharged home on subcutaneous insulin.

## Discussion

We describe a case of a new presentation of diabetes with both hyperosmolarity and ketoacidosis. Hyperosmolality and ketoacidosis occurs in approximately 30% of diabetic hyperglycaemic emergencies. Well-recognised complications include cerebral oedema, adult respiratory distress syndrome and vascular thrombosis, but this case illustrates two rarer complications: haemolysis and rhabdomyolysis.

The diabetic emergency revealed that this patient had previously undiagnosed G6PD deficiency. It was possible to diagnose G6PD deficiency by identifying it as a cause of both haemolysis and methaemoglobinaemia [1]. In a G6PD-deficient subject, haemolysis may occur as a result of ingestion of various drugs, infection and, more specifically, among diabetic subjects as a result of hypoglycaemia[2], blood glucose normalisation [3], ketoacidosis in the African[4,5] but not Mediterranean variant [6], and following administration of metformin [7] or glibenclamide [8]. The precipitant of the haemolysis is not clear in this case and may have involved a combination of infection, gliclazide and ketoacidosis.

It has been observed that defects in the G6PD gene correlate with diabetes and a more recent study proposed that alterations in genes controlling both insulin secretion and G6PD-mediated antioxidant defences may contribute to a predisposition to diabetes [9].

This patient also developed rhabdomyolysis. Very high glucose levels, high osmolality and low phosphate levels, which are all recognised risk factors for developing rhabdomyolysis, were demonstrated in this case. However, it is interesting to consider that G6PD muscle cell deficiency may also contribute to rhabdomyolysis [10].

## Conclusion

**Figure 2:**
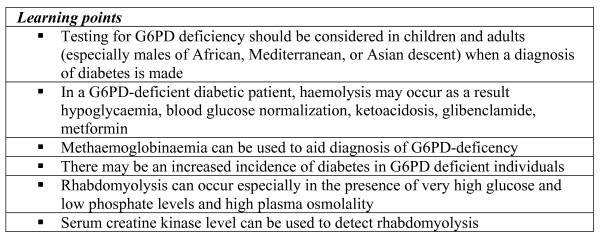
Summary of key learning points. This case has raised several important considerations when managing patients with G6PD deficiency and diabetes.

We have reported the case of a patient presenting with a diabetic crisis with hyperosmolarity and acidosis, which was complicated by haemolysis secondary to G6PD deficiency and rhabdomyolysis. This case highlights the importance of simple laboratory investigations, such as methaemoglobinaemia and serial measurements of creatine kinase (Figure 2), to detect these complications.

## Abbreviations

DKA: diabetic ketoacidosis; HONK: hyperosmolar nonketotic syndrome; G6PD: glucose-6-phosphate dehydrogenase.

## Competing interests

The authors declare that they have no competing interests.

## Authors' contributions

Both of the authors were involved in the writing of the manuscript and patient clinical care. Both authors read and approved the final manuscript.

## Consent

Written informed consent was obtained from the patient for publication of this case report and any accompanying images. A copy of the written consent is available for review by the Editor-in-Chief of this journal.

## Supplementary Material

Additional file 1 Renal function during the 22 days of admission. Renal function as demonstrated by creatinine levels. Normal values are shown by the grey lines. Click here for file
