# Supplementary figures and images for: New onset diabetes complicated by haemolysis and rhabdomyolysis: a case report and review of the literature
Source: J Med Case Rep. 2008 May 16;2:159. doi: 10.1186/1752-1947-2-159 (PMC2440389; doi:10.1186/1752-1947-2-159)

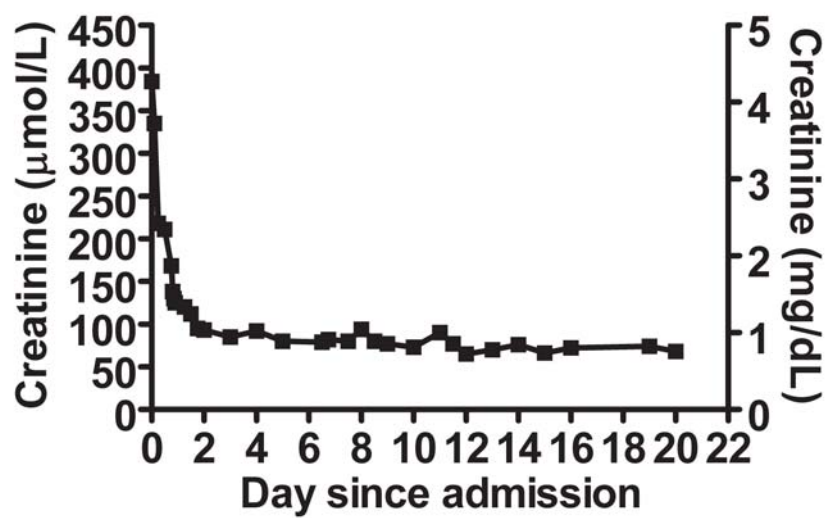

Supplement: Additional file 1 — Renal function during the 22 days of admission. Renal function as demonstrated by creatinine levels. Normal values are shown by the grey lines. [file 1752-1947-2-159-S1.pdf]
